# *Moringa oleifera* Hydroethanolic Extracts Effectively Alleviate Acetaminophen-Induced Hepatotoxicity in Experimental Rats through Their Antioxidant Nature 

**DOI:** 10.3390/molecules17078334

**Published:** 2012-07-10

**Authors:** Sharida Fakurazi, Syazana Akmal Sharifudin, Palanisamy Arulselvan

**Affiliations:** 1 Faculty of Medicine and Health Sciences, Department of Human Anatomy, Universiti Putra Malaysia, UPM Serdang 43400, Selangor, Malaysia; Email: chaz_kay86@yahoo.com; 2 Laboratory of Vaccines and Immunotherapeutics, Institute of Bioscience, Universiti Putra Malaysia, UPM Serdang 43400, Selangor, Malaysia; Email: arulbio@gmail.com

**Keywords:** antioxidants, N-acetylcysteine, liver markers, lipid peroxidation, hepatoprotection, total phenolic content

## Abstract

The aim of the study was to investigate the *in vitro* antioxidant properties *Moringa oleifera *Lam. (MO) extracts and its curative role in acetaminophen (APAP)- induced toxic liver injury in rats caused by oxidative damage. The total phenolic content and antioxidant properties of hydroethanolic extracts of different MO edible parts were investigated by employing an established *in vitro* biological assay. In the antihepatotoxic study, either flowers or leaves extract (200 mg/kg or 400 mg/kg, i.p) were administered an hour after APAP administration, respectively. N-Acetylcysteine was used as the positive control against APAP-induced hepatotoxicity. The levels of liver markers such as alanine aminotransferase (ALT) and the levels of oxidative damage markers including malondialdehyde (MDA), 4-hydroxynonenal (4-HNE) protein adduct, reduced glutathione (GSH), superoxide dismutase (SOD) and catalase (CAT) were analysed and compared between experimental groups. Among MO edible parts the flower extracts contain the highest total phenolic content and antioxidant capacity, followed by leaves extract. The oxidative marker MDA, as well as 4-HNE protein adduct levels were elevated and GSH, SOD and CAT were significantly decreased in groups treated with hepatotoxin. The biochemical liver tissue oxidative markers measured in the rats treated with MO flowers and leaves hydroethanolic extracts showed a significant (*p* < 0.05) reduction in the severity of the liver damage. The results of this study strongly indicate the therapeutic properties of MO hydroethanolic extracts against acute liver injury and thereby scientifically support its traditional use.

## 1. Introduction

Liver diseases are a major global concern and this type of disease/disorder still has extremely poor prognosis and high mortality because of the lack of effective preventive/treatment options. Despite remarkable advances in the field of modern medicine, hepatic diseases remain a major public health problem, thus the search for new effective medicines without side effects is still ongoing [1]. Hepatic cells are involved in a variety of metabolic events; therefore the establishment of liver protective/therapeutic agents is of paramount importance in the protection from liver damage. Natural remedies from traditional plants are seen as effective and safe alternative treatments for hepatotoxicity. The previous studies [2,3,4,5,6] have shown that hepatoprotective effects are associated with phytoextracts/phytocompounds rich in natural antioxidants. Many bioactive compounds and extracts from plants have thus been investigated for hepatoprotective and antioxidant effects against hepatotoxin-induced liver damage [7,8]. In addition, recent research on hepatoprotective medicinal plants as a major indicator of the screening systems can trigger the safety evaluation in the early phase of drug discovery because most of the toxic compounds are metabolized in liver [9,10,11,12].

Phenolic compounds are commonly found in both edible and other traditional medicinal plants, and they have been reported to have multiple biological activities, including free radical scavenging activity [13]. Antioxidants in food appear to play an essential role in the prevention of oxidative stress-related diseases/disorders and in the reduction of total mortality [14]. Typical phenolics that possess antioxidant activity are mainly phenolic acids and flavanoids. Flavonoids commonly accumulate in the epidermal cells of plant organs such as flowers, leaves, stems, roots, seeds and fruits, being found in glycosidic form (glycosides) and non-glucosidic form [15].

*Moringa oleifera* Lam is the most widely distributed species of the Moringaceae family throughout the World, especially in Asian countries, having a remarkable range of pharmacological properties in addition to significant nutritional value. *Moringa oleifera* is a highly valued plant in tropic and subtropical countries where it is mostly cultivated [16]. The medicinal properties of the plant’s edible parts have been recognized by both the Ayurvedic and Unani systems of medicine in India [17]. The various plant parts have wide medicinal applicability for the treatment of cardiovascular diseases as the roots, leaves, gum, flowers, and seed infusion contain nitrile, mustard oil glycosides, and thiocarbamate glycosides as their important bioactive constituents, which are thought to be responsible for their diuretic, cholesterol lowering, and antiulcer properties [18].

Atawodi *et al.* [19] showed the strong *in vitro* antioxidant properties of the methanolic extract of MO edible parts. This antioxidant activity of MO extracts is due to the presence of various bioactive compounds such as chlorogenic acid, rutin, quercetin glucoside, and kaempferol rhamnoglucoside. Furthermore, the extract of *Moringa* leaves and other parts have been shown to have potent antioxidant action *in vivo* [20,21,22,23].

Treatment with ethanolic extract of *M. oleifera* leaves or silymarin effectively protects hepatic tissues from tissue damage induced by antitubercular drugs [24]. Various studies in male rats have demonstrated the hepatoprotective effects of different MO edible part extracts against hepatotoxin-induced acute liver damage [25,26]. Moreover, our previous studies also showed that MO extract significantly protected the liver tissue against hepatotoxin-induced toxicity by enhancing the antioxidant defense system [5,27].

The leaves are highly essential nutritious when compared to other parts, being a source of protein, β-carotene, vitamins A, B, C and E, riboflavin, nicotinic acid, folic acid, pyridoxine, amino acids, minerals and various phenolic compounds [16,18]. The flowers also show effective hepatoprotective effects due to the presence of major amounts of the bioactive compound quercetin [28]. Seeds are used as a biosorbent for the removal of cadmium from aqueous medium and are one of the best-known natural coagulants discovered so far from natural sources [29]. They are also considered to be antipyretic, acrid, and bitter as well as reported to show antimicrobial activity [30].

To the best of our knowledge, the anti-hepatotoxic nature of MO flowers and leaves against APAP-induced liver injury in rats has not been demonstrated systematically. Hence, the present study focused on evaluating the anti-hepatotoxic potential of hydroethanolic extracts from MO flowers and leaves on APAP-induced liver toxicity in experimental rats. These results were compared with NAC (amino acid with antioxidant nature), a positive control drug for APAP induced hepatotoxicity studies.

## 2. Results and Discussion

### 2.1. Results

#### 2.1.1. Phenolic Compounds of MO Extracts

Total phenolics (TPC) in MO extracts were investigated for their total phenolic content (TPC) by the Folin-Ciocalteu assay. As shown in Table 1, the TPC values were expressed as mg gallic acid equivalents/g of dry extract (mg GAE/100 g dw) of the successive MO extracts. Ethanolic extracts showed higher phenolic content when compared to the aqueous extract. The ethanolic extracts from MO flowers showed the highest total phenolic content with 24.21 ± 1.55 mg GAE/100 g dw followed by leaves with 19.76 ± 0.26 mg GAE/100g dw.

#### 2.1.2. Antioxidant Property of Different Extracts of MO Investigated by their FRAP and DPPH Radical Scavenging Capacity (*in Vitro* Studies)

Table 1 shows the FRAP and DPPH assays, results were expressed as µM Trolox equivalent antioxidant capacity/100 g dry weight (µM TEAC/100 g dw). The ethanolic extracts were found to have higher antioxidant capacity compared to the aqueous extract of MO. The flowers extract has the highest total antioxidant capacity with 374.5 ± 3.1 µM TEAC/100g dw, followed by leaves with 237.6 ± 3.7 µM TEAC/100g dw. The results correlate well with the DPPH assay, where the flowers also showed the highest antioxidant capacity with 192.5 ± 4.1 µM TEAC/100 g, followed by leaves with 158.3 ± 5.3 µM TEAC/100 g dw.

**Table 1:** Phenolic content and antioxidant properties of *Moringa oleifera* hydroethanolic extracts.

	TPC (mg GAE/100 g dw)		FRAP (µM TEAC/100 g dw)		DPPH (µM TEAC/100 g dw)	
Parts\Solvent	Aqueous	Ethanolic	Aqueous	Ethanolic	Aqueous	Ethanolic
**Flowers**	17.11 ± 0.16	24.21 ± 1.55	315.2 ± 6.2	374.5 ± 3.1	170.8 ± 3.3	192.5 ± 4.1
**Leaves**	13.68 ± 0.23	19.76 ± 0.26	201.2 ± 8.1	237.6 ± 3.7	143.6 ± 6.1	158.8 ± 5.3
**Seeds**	5.66 ± 0.09	6.22 ± 0.28	180.1 ± 5.9	213.5 ± 2.4	104.1 ± 3.3	157.7± 5.2
**Pods**	8.37 ± 0.02	8.91 ± 0.16	143.3 ± 6.2	205.5 ± 8.6	88.3 ± 3.7	126.2 ± 7.7
**Stem**	2.16 ± 0.16	3.57 ± 0.07	121.1 ± 1.8	138.0 ± 4.8	14.7 ± 1.4	35.2 ± 1.2

*Abbreviation*s: TPC: Total phenolic content; FRAP: Ferric reducing antioxidant power; DPPH; 1,1-diphenyl-2-picrylhydrazyl; GAE: gallic acid equivalent; TEAC: Trolox equivalent antioxidant capacity. Values are mean ± SEM.

#### 2.1.3. Effect of MO Extracts on the Expression of 4-Hydroxynonenal Protein in APAP Induced Toxicity

**Figure 1:**
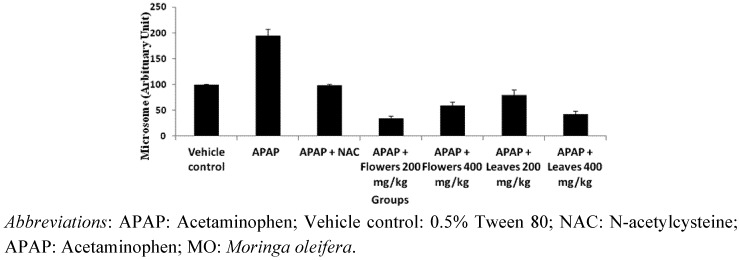
4-HNE adducted proteins observed following APAP, NAC and *Moringa oleifera* treatment. Hepatic tissues were collected from rats 24 h after administration with APAP. Image J analysis was performed and expressed in percentage (%). Animals treated with MO flowers and leaves extract shows declining 4-HNE adduct production.

The protein expression of 4-hydroxynonenal (4-HNE) protein adduct in liver microsomes by immuno-blotting analysis. As shown in Figure 1, there was an increase in the expression of 4-HNE protein adduct following administration of APAP to induce hepatotoxicity.

In the groups that were treated with flowers and leaves extracts, the expression of 4-HNE protein adduct was significantly reduced when compared with the APAP-induced toxicity group. The groups that were treated with vehicle control, the level of adduct was lower than in the groups treated with the hepatotoxin APAP.

#### 2.1.4. Effect of MO Extracts on Oxidative Stress Markers in APAP Induced Toxicity

Acetaminophen intoxication produces significant adverse effect on the redox status of the liver which was evidenced by biochemical tests. Malondialdehyde (MDA), a toxic product of lipid peroxidation was detected by thiobarbituric acid reactive substances analysis. Administration of APAP to experimental animals caused an increase in MDA production compared to the group administered with vehicle control. In groups that were treated with either 200 or 400 mg/kg flower extract showed significantly (*p* < 0.05) reduced MDA levels (0.41 ± 0.01 nM/mg protein and 0.46 ± 0.01 nM/mg protein), respectively. In addition, groups treated with MO leaves extract, there was also a reduction of MDA production, but it was not significant. At 200 mg/kg leaves extract, the MDA level was 0.51 ± 0.02 nM/mg protein (*p* < 0.05) and at 400 mg/kg the level was 0.43 ± 0.01 nM/mg protein (*p* < 0.05) (Table 2).

**Table 2:** Effect of MO flowers and leaves extract on hepatic MDA and GSH level in APAP induced hepatotoxicity in experimental rats. Values are presented as the means ± SEM of 6 rats per group. Statistical significance between the groups (^a ^*p* < 0.05;^ b^*p* < 0.05) compared to vehicle control and APAP treated groups respectively. Animal treated with 7 g/kg APAP showed significant changes of MDA production, GSH, SOD and CAT activity compared to appropriate controls. Administrating flowers and leaves extract one hour later shows animal recovering from oxidative stress.

Groups	MDA (nM/mg protein)	GSH (µM/mg protein)
Vehicle Control	0.42 ± 0.01	13.81 ± 0.24
APAP	0.61 ± 0.01^ a^	6.18 ± 0.12 ^a^
APAP + N-acetylcysteine	0.49±0.01^b^	9.39 ± 0.14^ b^
APAP + Flowers (200mg/kg)	0.41 ± 0.01^ b^	8.83 ± 0.06
APAP + Flowers (400mg/kg)	0.46 ± 0.01^ b^	9.70 ± 0.17 ^b^
APAP + Leaves (200mg/kg)	0.51 ± 0.02	7.13 ± 0.26
APAP + Leaves (400mg/kg)	0.43 ± 0.01^b^	8.89 ± 0.04^b^

Reduced glutathione, a non-enzymatic antioxidant thiol was reduced in liver homogenates of animals given APAP. Administration with flowers and leaves extract at a dosage of 400 mg/kg significantly (*p* < 0.05) increased the level of GSH to 9.70 ± 0.17 µM/mg protein and 8.89 ± 0.04 µM/mg protein, respectively. Neither 200 mg/kg flower extract nor 200 mg/kg leaves extract increased GSH levels significantly (*p* < 0.05, Table 2). These results show that flowers and leaves extract were able to preserve the amount of GSH, which is crucial to counteract the effects of APAP toxicity.

**Figure 2:**
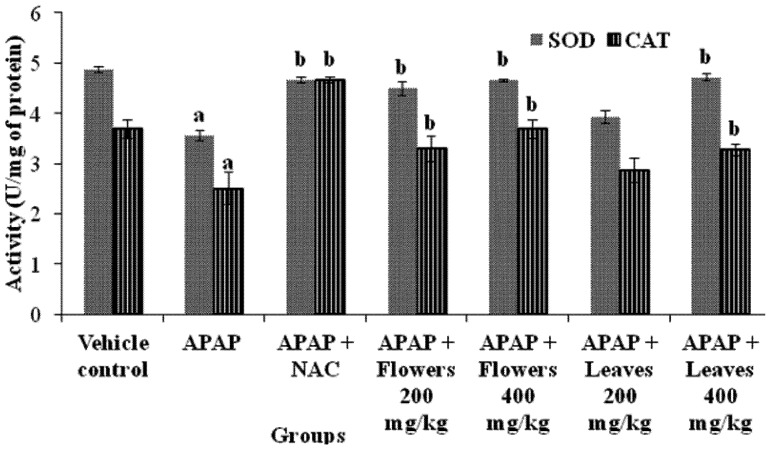
Effect of MO flowers and leaves extract on hepatic enzymatic antioxidants SOD and CAT in APAP induced hepatotoxicity in experimental rats. Values are presented as the means ± SEM of six rats per group. Statistical significance between the groups (^a ^*p* < 0.05;^ b ^*p* < 0.05) compared to vehicle control and APAP treated groups respectively.

The beneficial effects of MO extracts including flowers and leaves on the liver antioxidant system are presented in Figure 2. The activity of superoxide dismutase (SOD) was significantly (*p* < 0.05) decreased in APAP intoxicated rats. Superoxide dismutase is known to be the primary defense system against oxidative stress. Catalase (CAT) is one of the most important intracellular enzymes in the detoxification of the oxidant hydrogen peroxide. The activity of these enzymes was inhibited due to high level of toxic metabolites. After administration of flowers extract, the activity of these enzymes was increased significantly (*p* < 0.05) for different dose. As for the leaves extract, the influence of antioxidant enzyme activity was dose-dependent.

#### 2.1.5. Effect of MO Extracts on Liver Function

**Figure 3:**
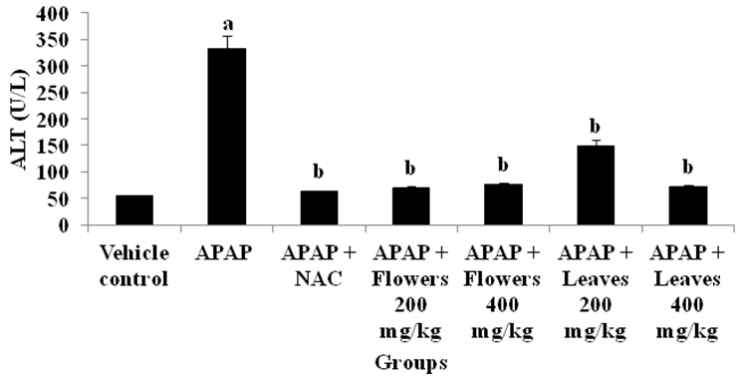
Effect of MO flowers and leaves extract on hepatic ALT levels in APAP induced hepatotoxicity in experimental rats. Values are presented as the means ±SEM of six rats per group. Statistical significance between the groups (^a ^*p* < 0.05; ^b ^*p* < 0.05) compared to vehicle control and APAP treated groups respectively.

The activity of ALT used as biochemical markers for evaluation of early hepatic injury, were significantly increased in the hepatotoxin, APAP treated rats (Figure 3).

Meanwhile, animals that were treated with N-acetylcysteine showed significant (*p* < 0.05) reduction of ALT and AST level (data not shown). In groups that were treated with extracts from flowers and leaves, the activity of liver marker enzymes were significantly (*p *< 0.05) reduced when compared with the appropriate control.

### 2.2. Discussion

Phenolic compounds are known for their potent antioxidant properties. This class of plant metabolites contains more than eight thousand known compounds, ranging from simple phenol to complex materials such as tannins. Balasundram *et al.* [31] showed that phenolics are the main dietary antioxidants and possess higher *in vitro* antioxidant capacity than the essential vitamins and carotenoids. Various findings have also shown that the phenolic-rich hydroethanolic fractions of plants contain catechin, rutin, quercetin, kaempherol and isorhemnetin [32]. In this present study, it is shown that MO flowers have the highest total phenolic content and antioxidant capacity than other MO parts. The presence of the active phenolic compound quercetin, a powerful antioxidant, in the flowers was reported by Ruckmani *et al. *[33]. These findings were correlated with previous studies [22] mainly concerning phenolic compounds and their antioxidant properties.

The MO leaves contain different classes of phytocompounds, vitamins and carotenoids [34] and these compounds mainly contribute to the antioxidant properties as well as other biological activities. β-Carotene from MO leaves is efficiently converted into vitamin A in the body and has shown significant hepatoprotective effects. These reported studies have strongly suggested the importance of phenolic compounds from MO and their antioxidant properties. The main antioxidant activity that has been associated with phenolic content ability is to scavenge free radical formation [22]. Certain phenolic compounds also may induce production of glutathione-S-transferase and other antioxidant enzymes. The ability of certain phenolic compound to bind to minerals may be beneficial in some cases, since copper and iron can be initiators of hydroxyl radical production by the Fenton and Haber-Weiss reactions [35].

Yen *et al.* [36] showed that treatment with hydroethanolic extract of *Cuscuta chinensis* prevented the progression of liver damage due to hepatotoxins in an APAP-induced experimental system. It enhanced the activity of antioxidant enzymes and diminishes the amount of toxic lipid peroxides. However the aqueous fraction of the plant did not ameliorate the histopathological changes resulting from APAP-induced hepatotoxicity, suggesting the hydroethanolic fraction contains phenolic compounds with strong antioxidant properties. The amount of phenolic compounds was also dependent on the different extraction method [37]. These results were consistent with previous studies described on antioxidant properties of MO extracts, mainly phenolics [23].

Acetaminophen (APAP, 4-hydroxyacetanilide) is a well-known antipyretic and analgesic agent, which is harmless in therapeutic doses but can produce fatal hepatic necrosis in experimental animals as well as humans [38] and is employed as a well-established experimental hepatotoxic agent for pre-clinical research. The availability of APAP in pharmacies without prescription has led to it being kept in many homes and it is, therefore, not surprising that it is regularly involved in episodes of accidental and deliberate self poisoning [39]. The drug is remarkably safe with an excellent safety record in terms of unwanted side effects. However, accidental or intentional APAP overdosage could cause life threatening liver damage and associated diseases.

At limited therapeutic doses, APAP is metabolized by cytochrome P450 (CYP) to form the highly reactive species, *N*-acetyl-*p*-benzoquinone imine (NAPQI), which under normal conditions is readily detoxified by conjugation with glutathione (GSH). However, in human and experimental animals, high doses of APAP saturate detoxification pathways, leading to hepatic glutathione depletion and excessive production of NAPQI, which freely binds to cellular metabolites/molecules [40].

Adduction and modification of functional and signaling proteins are likely to be one of the mechanisms by which 4-HNE can influence physiologically as well as pathological processes [41]. Once formed, HNE is rapidly degraded by three major reactions: reduction by alcohol dehydrogenase, and aldehyde dehydrogenase or formation of glutathione conjugate (GS-HNE) catalyzed by GST. The majority of HNE is metabolized through forming GS-HNE [42]. However, HNE impairs cellular function by 1,4-Michael addition attachment to specific amino acids including cysteine and lysine [43].

In this study, the level of 4-HNE protein adducts were higher in animals that were given APAP alone. The 4-HNE adducts were reduced after the extracts administration and this may suggest that the plant extracts may be partly involved in HNE metabolism to counteract the APAP-induced toxicity. Animals treated with NAC showed similar adduct reduction patterns compared to the groups treated with the extract. Uma *et al*. [27] reported that GST levels were increased in rats treated with MO leaves extract. Therefore, GST may play a critical role in hindering the formation of 4-HNE adducts with cellular macromolecules. This finding suggest that HNE adduct might have undergone glutathione conjugation process which is catalysed by GST. This results coincides with our findings where in animals treated with plant extracts, the HNE adduct expression was reduced whilst the level of GSH and GST were increased significantly (Figure 1).

Reactive oxygen species (ROS) are related to oxidative stress and many scientific reports have shown that excessive production of ROS can further aggravate oxidative stress and have implicated ROS in a number disorder/disease processes, including heart disease, diabetes, liver injury, cancer, and aging [44,45,46]. Equilibrium between ROS and enzymatic antioxidant enzymes including superoxide dismutase (SOD), catalase (CAT), and glutathione peroxidase (GPx) are crucial and could be an important mechanism for preventing damage by oxidative stress. The liver plays a central role in the maintenance of systemic lipid homeostasis and is especially susceptible to ROS damage. This mechanism has been suggested to play role in preventing APAP toxicity [47].

Natural antioxidants and other synthetic or natural ROS scavengers may reduce the incidence of free radical-mediated diseases. The use of antioxidants in the prevention and treatment of various disorder/diseases is intensifying, and there is considerable clinical interest in the study of the antioxidant activities of molecules such as plant polyphenolic and carotenoid components [48,49]. Antioxidants appear to act against disease processes by increasing the levels of endogenous antioxidant enzymes and decreasing toxic products such as lipid peroxidation byproducts [50]. MO extracts have strong antioxidant properties due to the various bioactive constituents found in different solvent extracts [22]. These antioxidant bioactive compounds effectively prevent liver damage from hepatotoxin-induced toxicity.

Malondialdehyde (MDA) is one of the end-products of polyunsaturated fatty acid peroxidation and is a good indicator of the degree of lipid peroxidation [51], which is related to APAP-induced tissue damage. In the present study, a significant increase in the MDA level observed in liver homogenate of APAP-intoxicated rats was reduced by treatment with the MO flowers and leaves, indicating its ability to break the chain reaction of lipid peroxidation. Based on these results, we may suggest that the therapeutic potential of MO extracts is dependent on an antioxidant mechanism. These results concluded that MO extracts effectively inhibit APAP-induced tissue damage due to presence of various antioxidant bioactive compounds (Table 2).

Glutathione is an important nonenzymatic antioxidant that protects the liver against acetaminophen-induced damage [52]. The depletion of cellular GSH level in the hepatic cells is known to play a key role in APAP toxicity [53]. APAP administration leads to a significant decrease in the reduced glutathione concentration which can be an important factor in the APAP induced toxicity. Our study has shown that the level of GSH was restored following 400 mg/kg extracts administration to the nearly same concentration of NAC suggesting that MO flowers and leaves have some beneficial role in order to improve glutathione pathway (Table 2). The mechanism of therapeutic action by MO extracts against APAP toxicity might be due to restoration of the GSH level and presence of bioactive compounds. These results were consistent with our previous findings on hepatoprotective nature of MO extracts [5].

Superoxide production comes from various sources such as autooxidation of leukoflavins and the products of mitochondria. In human diseases superoxide is produced by the aerobic oxidation of purines by xanthine oxidase [54]. The role of superoxide dismutase depletion in the pathogenesis of APAP intoxication was supported by various studies performed in different experimental models [55,56]. A decrease in SOD production can be attributed to an enhanced superoxide generation and utilization of this enzyme during reactive metabolites detoxification. 

High amounts of superoxide inhibits CAT, which is another important antioxidant enzyme [57]. Catalase, that decomposes hydrogen peroxide and protects tissue from reactive hydroxyl radicals is widely distributed in all animal tissues. Administration of APAP alone significantly reduced SOD and CAT activity, indicating oxidative stress. Upon administration of flowers and leaves extract, the superoxide toxicity was diminished as SOD and CAT levels were increased significantly suggesting either the plant itself reacts with the reactive oxygen species or boosting the antioxidant enzyme production. Catalytic removal of reactive oxygen species by SOD and CAT enzymes are extremely important as they prevent numerous lipid peroxidation by-products, protein adduction and organ dysfunction. Moreover, these findings support the beneficial effect of MO extracts in maintaining the hepatocytes integrity and metabolic function. Based on the previous plant metabolite profiles of *M. oleifera* extracts, the have high amounts of phenolic compounds and flavonoids, which are known to effectively scavenge free radicals. It is conceivable that these effects may be due, at least in part, to its higher antioxidant activity (Figure 2).

Liver function tests help in the diagnosis of any abnormal/normal condition of liver. Leakage of cellular enzymes into plasma indicates the sign of hepatic tissue damage [58,59]. Generally measurement of alanine aminotransferase (ALT) is used as an important diagnostic marker to indicate liver injury due to hepatotoxins.

Administration of MO flowers and leaves extract significantly reduced the extent of liver damage following a high dose of APAP (Figure 3). There was a significant decrease of serum ALT level in rats treated with flowers and leaves extract as well, as N-acetylcysteine, and these results are correlated with other research findings [60,61]. This result showed that there was a reduction in the damage of parenchymal and mitochondrial sites of the liver where ALT is located. This proved that the integrity of the liver cells was preserved from leakage as ALT enzymes were reduced in blood stream. These results proved that MO extracts significantly decreased liver toxicity due to its nontoxic nature and tissue protective nature against various toxic metabolites.

## 3. Experimental Section

### 3.1. Drugs and Chemicals

Acetaminophen (APAP) was purchased from Sigma Aldrich Co., (France) and absolute ethanol were from Fisher Scientific (USA). Nitroblue tetrazolium chloride, potassium chloride and sodium chloride were from Merck (Germany). Sodium hydroxide, 5,5,-dithiobis-2-nitrobenzoic (DTNB) (Fluka, Australia) 1,1,-diphenyl-2-picrylhydrazyl (DPPH), phenazine methosulphate, potassium dichromate (K_2_Cr_2_O_7_), thiobarbituric acid (TBA), trichloroacetic acid (TCA), 2,4,6-tripyridyl-*s*-triazine (TPTZ) and 6-hydroxy-2,5,7,8-tetramethylchroman-2-carboxylic acid, were procured from Sigma (Germany). The diagnostic kit for alanine aminotransferase (ALT) was obtained from the Roche Diagnostics (Germany). All other chemicals used were of analytical grade (AR).

### 3.2. Plant Material and Preparation of Extract

The plant materials (leaves, flowers, seeds, pods and stem) were identified and harvested in Taman Pertanian Universiti (TPU), Malaysia and verified by a plant taxonomist. Mr. Shamsul Khamis, Institute of Bioscience (IBS), Universiti Putra Malaysia and a voucher specimen (SK 1561/08) has been deposited at IBS Herbarium unit for future reference. Samples were soaked and occasionally agitated in 80% ethanol for six days. All samples were then filtered with a double layer of gauze before using filter paper. The samples were then concentrated and lyophilized and kept at −20 °C for further experiments [5]. Samples were aliquoted with 0.5% Tween 80 for further experiments.

### 3.3. Animals

Male Sprague-Dawley rats were acclimatized for one week in the animal house (26 ± 2 °C) with 12 h light and dark cycles. They were fed with standard rodent pellet and water was provided *ad libitum*. All animal experiments were approved (Approval No: UPM/FPSK/PADS/BR-UUH/00356) and conducted by IACUC (Institutional Animal Care and Use Committee), Faculty of Medicine and Health Sciences, Universiti Putra Malaysia, Malaysia.

### 3.4. Quantification of Phenolic Compounds and *in Vitro* Antioxidant Assay of MO Extracts

Samples were grouped according to the different parts of the plant material and they were compared between methods of extraction. In the animal study, hydroethanolic extract was dissolved in 2.0 mL of 0.5% Tween 80 as vehicle.

### 3.5. Estimation of Total Phenolic Content

The assay was conducted according to Wong *et al.* [62] with slight modification. Aliquots (0.5 mL) of plant extracts were mixed with 10% (v/v) Folin-Ciocalteu reagent (2.5 mL) and 7.5% (w/v) sodium carbonate (2.0 mL). The mixture was left at room temperature for 1 h. Absorbance of the resulting blue colour was measured at 765 nm using a Shimadzu UV-VIS spectrophotometer. The results were expressed as gallic acid equivalent (GAE) per 100g of dry weight.

### 3.6. DPPH Free Radical Scavenging Activity

The DPPH assay was determined using the method proposed by Yen and Chen [63] and Wojdylo *et al.* [64] by preparing DPPH solution (0.45 mM) in absolute ethanol (99%). Extracts (0.5 mL) were added with DPPH (1.0 mL) and allowed to react for half an hour in the dark place. The decrease in absorbance of the result for the solution was monitored at 517 nm. A standard curve was prepared using different concentrations of Trolox.

### 3.7. Ferric Reducing Iron Power (FRAP) Assay

The antioxidant potential of phytoextracts were determined using the FRAP assay described by Benzie and Strain [65] and Wojdylo *et al.* [64]. A potential antioxidant reduces ferric ion (Fe^3+^) to the ferrous ion (Fe^2+^). The ferric ion reducing power reagent consists of 10 µM TPTZ in 40 µM HCl, 20 µM ferric (III) chloride and 30 mM acetate buffer pH 3.6 (1:1:10). Extract (0.1 mL) was added to distilled water before adding FRAP reagent (3.0 mL). Absorbance was read at 593 nm at 5 min. A standard curve was prepared using different concentrations of Trolox.

### 3.8. Therapeutic Study against Hepatotoxin Induced Liver Toxicity

Male Sprague-Dawley rats (200–250 g) were randomly assorted into groups of six animals (n = 6). Acetaminophen was dissolved in vehicle and given via oral route. In this present study, one hour after APAP intoxication, MO extracts and NAC positive control drug were administered via intraperitoneal route.

### 3.9. Experimental Design

Group I: Animal received 0.5% Tween 80 only (vehicle control).

Group II: Animals received 7 g APAP/kg bw.

Group III: Animals received 7 g APAP/kg bw and 7.35 mmol N-acetylcysteine/kg bw [66] (positive control).

Group IV: Animals received 7 g APAP/kg bw and 200 mg flowers extract/kg bw.

Group V: Animals received 7 g APAP/kg bw and 400 mg flowers extract/kg bw.

Group VI: Animals received 7 g APAP/kg bw and 200 mg leaves extract/kg bw.

Group VII: Animals received 7 g APAP/kg bw and 400 mg leaves extract/kg bw.

Diet was withdrawn from the animals 16 h before sacrifice. Animals were weighed and sacrificed 24 h after the treatment using diethyl ether. Blood was collected via cardiac puncture for biochemistry analysis. Liver was removed from rats and weighed. The serum and liver samples were stored at −80 °C until further analysis.

### 3.10. Preparation of Homogenates, Cytosol and Microsomal Fractions

Liver was weighed and homogenized in ice cold 0.15 M Tris-KCl buffer (0.15M KCl + 10 mM Tris-HCl, pH 7.4). The homogenate was then centrifuged at 600 g for 10 min. Supernatant was collected and centrifuged again at 10,000 g for 30 min. The mitochondrial fraction was collected and re-suspended in sucrose buffer whereas the supernatant was centrifuged at 105,000 g for 60 min to obtain the cytosolic and the microsomal fractions. Liver subcellular fractions were kept under −80 °C until further studies. Protein content was determined using Bradford [67] method using bovine serum albumin (BSA) as a standard.

### 3.11. 4-Hydroxynonenal (4-HNE) Protein Adduct

The method was conducted according to Terneus *et al.* [66]. Microsomal fraction of the liver was used in this experiment. Samples and standards were run on 12.5% acrylamide polyacrylamide gel for 70 min at 30 mA/gel. 4-Hydroxynonenal protein adduct was detected using rabbit polyclonal (E)-4-hydroxynonenal antibody (anti-HNE PAb) (1:1000 Alexis Biochemicals, ALX-210–767, Sigma, USA). Incubation with primary antibody was done overnight under constant shaking at 4 °C. The following day the membrane was incubated with goat anti-rabbit horseradish peroxidase (HRP)-conjugated secondary antibody (1:3000 Serotec) for 2 h. The membrane was developed using enhanced chemiluminescent substrate (Thermo Scientific, USA) for visualisation of 4HNE-protein adduct. The developed membrane was exposed using GelDoc (Chemismart-3126 WL/26MX, France) for 2–20 s. Protein bands were analysed using Image J (version 1.0) software. The final data was corrected with β-actin factor to normalise the differences between each sample loading volume.

### 3.12. Determination of Malondialdehyde (MDA) Level

Lipid peroxidation in the liver was ascertained by formation of malondealdehyde (MDA) and measured by thiobarbituric reactive (TBARS) method previously described by Onkawa *et al.* [68]. Reaction mixture containing homogenate (0.5 mL), TCA (0.5 mL) and TBA (0.5 mL) was incubated in boiling water for 15 min. The pink colour of chromogen formed was extracted in butanol solution (2.0 mL). The mixture was centrifuged at 3000 rpm for 10 min and the supernatant was read at 532 nm.

### 3.13. Determination of Reduced Glutathione (GSH) Level

The GSH level was determined using method previously by Ellman [69] with slight modifications. Homogenate (0.2 mL) was added with 25% TCA and centrifuged at 3000 rpm for 10 min. Supernatant (0.2 mL) was added with 10 mM of DTNB in the presence of phosphate buffer (0.1 M, pH 7.4). Absorbance was read at 420 nm.

### 3.14. Determination of Superoxide Dismutase (SOD) Activity

Cytosol fraction of the liver was used in this assay as previously described in Kakkar *et al.* [70]. The cytosolic fraction (0.05 mL) was added with sodium pyrophosphate buffer (0.052 M, pH 8.3, 1.2 mL), phenazine methosulphate (0.186 mM, 0.1 mL), nitroblue tetrazolium chloride (0.3 mM, 0.3 mL), and NADH (0.78 mM, 0.2 mL). Reaction was stopped after 90 s with glacial acetic acid. Colour intensity of the chromogen was extracted in butanol solution (2.0 mL) and shake vigorously. The mixture then was centrifuged at 3000 rpm for 10 min and the supernatant was measured at 560 nm.

### 3.15. Determination of Catalase (CAT) Activity

The catalase enzyme activity in tissues was assayed following the procedure of Sinha [71]. Homogenate (0.1 mL) was incubated with H_2_O_2_ (0.2 M, 0.5 mL), in the presence of 0.01 M phosphate buffer (pH 7.4). Reaction was stopped by adding 5% dichromate solution. Samples were incubated in boiling water for 15 min. Phosphate buffer (2.0 mL) was added and shaken vigorously. The upper layer of the mixture was taken and the absorbance read at 570 nm.

### 3.16. Determination of ALT Level

Activity of ALT level was estimated by commercial kinetic method kits from Roche Diagnostics (Germany) according to the standard protocol.

### 3.17. Statistical Analysis

All the data are presented as mean ± SEM. Statistical analysis was performed using Statistical Package for the Social Sciences (SPSS) version 16.0. Statistical significance of the differences between the treatments groups in experiments were analysed by post-test Tukey’s multiple comparison tests. *p* < 0.05 was mentioned in the results as statistically significant.

## 4. Conclusions

The present investigation suggested that MO has a potential role in therapeutic action via inhibiting oxidative stress due to presence of phenolic compounds and its anti-oxidant nature. However, further investigations are essential to elucidate the precise molecular mechanism of specific bio-active agents from MO leaves and flowers for protection/treatment against hepatotoxin induced hepatotoxicity and it has to be tested against various biologically important markers.
